# A consensus guideline for antipsychotic drug use for dementia in care homes. Bridging the gap between scientific evidence and clinical practice

**DOI:** 10.1017/S1041610215000745

**Published:** 2015-06-10

**Authors:** Sytse U. Zuidema, Alice Johansson, Geir Selbaek, Matt Murray, Alistair Burns, Clive Ballard, Raymond T. C. M. Koopmans

**Affiliations:** 1 Department of General Practice, University Medical Center Groningen, University of Groningen, Groningen, the Netherlands; 2 Department of Primary and Community Care, Centre for Family Medicine, Geriatric Care and Public Health, Radboud University Medical Centre, Nijmegen, the Netherlands; 3 Norwegian National Advisory Unit of Ageing and Health, Vestfold Hospital Trust, Tønsberg, Norway; Centre for Old Age Psychiatry Research, Innlandet Hospital Trust, Ottestad, Norway; Institute of Clinical Medicine, Campus AHUS, University of Oslo, Norway; 4 UK Alzheimer's Society, London, UK; 5 Faculty of Medical and Human Sciences, Institute of Brain, Behaviour and Mental Health, The University of Manchester, UK; 6 Wolfson Centre for Age-Related Diseases, Kings College London, UK; 7 Department of Primary and Community Care, Centre for Family Medicine, Geriatric Care and Public Health, Radboud University Medical Centre, Nijmegen, the Netherlands and Joachim & Anna, center for specialized geriatric care, Nijmegen, the Netherlands

**Keywords:** dementia, psychopharmacology, nursing homes

## Abstract

**Background:**

To produce a practice guideline that includes a set of detailed consensus principles regarding the prescription of antipsychotics (APs) amongst people with dementia living in care homes.

**Methods:**

We used a modified Delphi consensus procedure with three rounds, where we actively specified and optimized statements throughout the process, utilizing input from four focus groups, carried out in UK, Norway, and the Netherlands. This was done to identify relevant themes and a set of statement that experts agreed upon using the Research and Development/University of California at Los Angeles (RAND/UCLA) methodology.

**Results:**

A total of 72 scientific and clinical experts and 14 consumer experts reached consensus upon 150 statements covering five themes: (1) General prescription stipulations, (2) assessments prior to prescription, (3) care and treatment plan, (4) discontinuation, and (5) long-term treatment.

**Conclusions:**

In this practice guideline, novel information was provided about detailed indication and thresholds of symptoms, risk factors, circumstances at which APs should be stopped or tapered, specific criteria for justifying long-term treatment, involvement of the multidisciplinary team, and family caregiver in the process of prescription. The practice guideline is based on formal consensus of clinicians and consumer experts and provides clinicians relevant practical information that is lacking in current guidelines.

## Introduction

APs are widely prescribed for the treatment of neuropsychiatric symptoms (NPS) for people with dementia, especially in long-term care settings. People with dementia in care homes have a higher risk of AP drug prescription compared to those residing in the community (Maquire *et al.*, 2013). Prescription rates range from 20 to 50% with some variation among countries (Feng *et al.*, 2009, Wetzels *et al.*, 2011, Barnes *et al.*, 2012), and between nursing homes (Selbaek *et al.*, 2008, Zuidema *et al.*, 2011), This variation is probably largely explained by differences in clinical and care practice (Zuidema *et al.*, 2011; Cornegé-Blokland *et al.*, 2012). Moreover, APs are often used for prolonged periods of >6 months (Wetzels *et al.*, 2011, Barnes *et al.*, 2012, Gustafsson *et al.*, 2013), sometimes without an appropriate ongoing indication/regular review (Chen *et al.*, 2010) highlighting non-adherence to current guidelines (Morley, 2012).

APs have a modest but significant benefit over a period of 12 weeks in the treatment of both aggression and psychosis, but also widely reported adverse events, like extrapyramidal symptoms, sedation, falls, accelerated cognitive decline, and increased risk of stroke, pneumonia, and a 1.5–1.7 fold increased risk of mortality (Gareri *et al.*, 2014). APs can be withdrawn without relapse of NPS (Declerq, *et al.*, 2013), although a small proportion of people do have a return of symptoms with cessation or reduction of APs (Ballard *et al.*, 2009a; Devanand *et al.*, 2012). Moreover, there is evidence that psychosocial interventions may help to reduce AP drug use (Richter *et al.*, 2012).

Although prescription rates tend to decrease in some countries (Health and Social Care Information Centre, National Dementia and Antipsychotic Prescribing Audit, 2012; Schulze *et al.*, 2013), there is still a long way to go in reducing use of APs and convince physicians towards more appropriate use. Many physicians believe that existing evidence-based guidelines are not adequate for daily practice (McCleery and Fox, 2012). Therefore, there is a need to fill the gap between the existing evidence and daily practice and to develop more practice-based recommendations for appropriate prescription of APs.

Some key topics in the prescription of APs are not sufficiently addressed in the consensus papers and other practice guidelines such as the American Geriatrics Society and American Association for Geriatric Psychiatry (AGS/AAGP) consensus statement (AGS/AAGP, 2003), the Canadian guideline for seniors’ mental health (Conn *et al.*, 2006), the National Institute for Health and Care Excellence (NICE) guideline Dementia for supporting people with dementia and their carers in health and social care (NICE clinical guideline 42, 2006), and the Behavioral and Psychological Symptoms in Dementia (BPSD) guide (British Alzheimer's Society, 2011). These topics include: (1) detailed indication and thresholds to prescribe APs in agitation, aggression, and psychosis, (2) risk factors that should be considered before prescription, (3) circumstances at which APs should be stopped or tapered, (4) specific criteria for justifying long-term treatment, (5) involvement of the multidisciplinary team and family caregiver in the process of AP prescription.

Previous practice guidelines mentioned above has largely relied on so-called type IV evidence. The decision process that has led to this kind of consensus statements are not clearly prescribed in previous consensus papers and practice guidelines, may depend on the experts’ own individual preferences and lacked the direct involvement of consumer experts.

The aim of this study was to produce a practice guideline that includes a set of detailed principles regarding the prescription of APs amongst people with dementia living in care homes. The guideline had to be agreed upon by scientific, clinical, and consumer experts using a modified Delphi consensus procedure and really contribute to the key topics of gaps in scientific knowledge mentioned above.

## Methods

### Description of the modified Delphi consensus procedure

We combined a Delphi consensus procedure of three iterations (adapted from Hsu and Sanford, 2007) in which experts rated their agreement with predefined statements in a series of structured questionnaires with two phases of focus groups where respondents discussed the items/statements.

**Figure 1.**
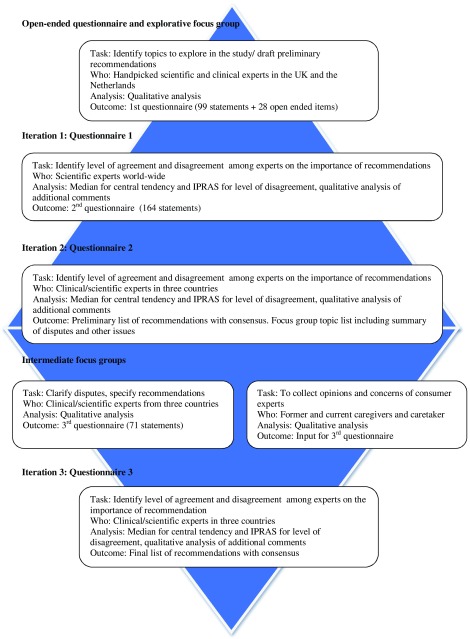
Delphi process.

The research team (AJ, SZ, CB, RK, MM) provided the topic lists of the focus groups and proposed changes of the questions/statements at each consecutive Delphi round. Scientific experts, clinical experts, and consumer experts participated in the Delphi iterations/the focus groups and responded to the topics/statements. A scientific expert was defined as “someone who has published several national or international papers on APs in care homes.” A clinical expert was defined as “someone who works in the field of long-term care and/or is familiar with current literature on AP drug use”. A consumer expert was defined as “a (former) caregiver with experience with or interest in APs, or people with a diagnosis of (mild) dementia.” This modified Delphi procedure consisted of five phases (Figure 1).

1. An explorative focus group interview with 20 scientific and clinical experts based on open-ended questions, in order to generate ideas and identify core issues.
2. Iteration 1/Questionnaire 1: based on the input of the exploratory focus group, a first structured questionnaire was sent out by email to 127 scientific experts worldwide. The response on the statements that could be agreed upon or not on a 5-point Likert scale was used to further refine the questionnaire for iteration 2.
3. Iteration 2/Questionnaire 2: a second questionnaire was sent to 46 scientific/clinical experts in the UK, the Netherlands and Norway with 164 statement covering eight themes, that could be agreed upon or not on a 9-point Likert scale (see Table 1). We also developed a separate questionnaire for the consumer experts. This questionnaire consisted of 54 statements, derived from the larger questionnaire for the scientific/clinical experts and covered four themes: (1) NPS that may justify the treatment of APs, (2) provisions that should be in place before prescription is justified, (3) the threshold at which prescription is justified, (4) consultation with family caregiver about AP treatment. Statements that were agreed upon were entered in the practice guideline. Statements that were not agreed upon were discussed in the clinical experts and consumer experts focus groups.
4. Intermediate focus groups: four focus groups were organized: three with clinical experts (one at each country) and one with consumer experts from the UK. The consumer experts were recruited via Alzheimer's Society's Network in the UK. The themes of the focus groups were based on the controversies of the second questionnaire. The input from the focus groups was used to specify the statements further and to formulate some new ones.
5. Iteration 3/questionnaire 3: the third questionnaire was developed in the same format as the previous and was sent out to the same experts, who participated in iteration 2. In the final phase of the study, we collected all statements on which there was agreement. These were summarized into a recommendation guideline.

**Table 1.** Response rate and professional division of respondents

iteration	focus group	iteration 1	iteration 2	focus group	iteration 3
Total number	*n* = 15	*n* = 40	*n* = 34	*n* = 21	*n* = 33
Geographical coverage	NL, UK	International	NL, N, UK	NL, N, UK	NL, N, UK
*Key professional expertise*					
(Old Age) Psychiatrist	10	29	14	13	15
Neurologist	–	2	–	–	–
Geriatrician	–	3	8	2	8
Elderly care physician	2	–	–	3	–
Nurse	–	1	1	1	1
Nurs home doctor/GP	–	2	2	1	2
Scientist	–	3	–	–	–
Policy maker	2	–	–	–	–
Clin Pharmacologist	–	–	5	–	4
Spec fam. medicine	1	–	2	–	2
Clinical Psychologist	–	–	1	1	1
Pharmacist	–	–	1	–	–

^a^The focus group of consumer experts was organized in the UK and consists of six former caregivers, two current caregivers and one person with dementia.

### Analysis

We used two criteria, specified in the RAND/UCLA appropriateness method, for measuring the level of agreement and determine consensus; namely the median rating and the inter-percentile range (IPR) adjusted for symmetry (IPRAS). The median was calculated to measure central tendency. IPRAS was calculated to measure the level of dispersion of the ratings. IPRAS is the threshold beyond which the IPR for a particular item indicates disagreement. Statements with a median between 7 and 9, and on which there was agreement, when the IPRAS was controlled for, were included in the guideline, unless additional comments motivated further specification or clarification.

The focus group discussions were recorded and transcribed verbatim to capture the exact words and phrases of the participants. The transcriptions were then analysed qualitatively by analyzing the content of the topics and grouping similar arguments together.

## Results

### Participants involved

A total of 40 respondents participated in the first iteration (31% of those invited). In the second iteration, a total of 34 experts participated (74% of those invited). In the third iteration, 31 experts participated (89% of those invited). Professionals were (old age) psychiatrists, neurologists, geriatricians, elderly care physicians (Koopmans *et al.*, 2010), a GP, nurses, clinical pharmacologist, and a clinical psychologist. Of the 34 respondents in the second iteration, 21 participated in the focus group (5 in the Netherlands, 10 in Norway, and 6 in the UK). Fourteen consumer experts with experience with APs (7% of the 196 invited) agreed to participate in the second iteration, of which 9 participated in the intermediate focus group and the third iteration. Six of these were former caregivers, 2 were current caregivers with a parent who had been prescribed APs. One participant was diagnosed with dementia, but was not prescribed AP medication (Table 1).

### Agreement on statements

**Figure 2.**
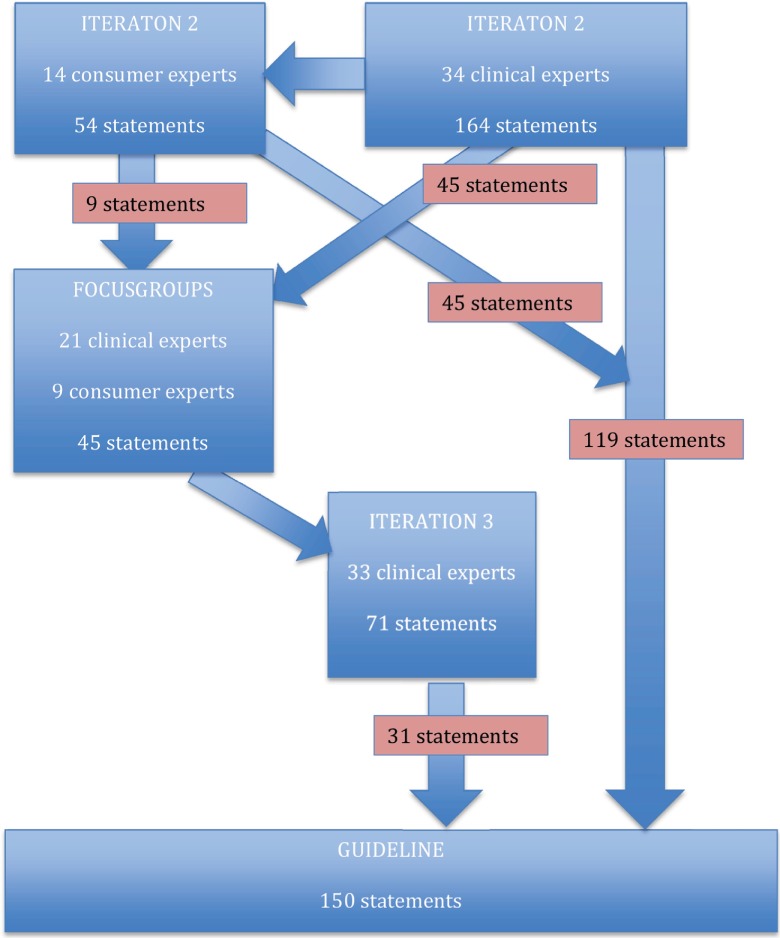
Origin and refinements process of the statements during Delphi round iteration 2, intermediate focus group, and iteration 3.

Respondents reached agreement with median values between 7 and 9 on 119 of 164 statements in the second and 31 out of 71 statements in the third questionnaire. The third questionnaire was obviously shorter because items that were agreed upon were left out of the next questionnaire. The consumer experts questionnaire consisted of 54 statements of which 15 were specifically added by this group. The consumer experts reached consensus on 45 out of 54 statements, including 9 out of the 15 statements that were brought up by this group. In total, the respondents reached agreement on 150 statements with median score between 7 and 9 that were included into the guideline. (Table 2). During the Delphi process and focus group some statements that were not agreed upon were further refined, of which some of them reached final agreement. Examples were as follows: electrocardiogram was advised before prescription only in patients with increased cardiovascular risk, APs should not always be prescribed in cases of acute aggression but only when patient's behavior cause acute danger to themselves, other patients or other people. The origin and the refine process of the statements are depicted in Figure 2.

**Table 2.** Results iteration 2 and 3

iteration	iteration 2	iteration 3
**Number of statements**	*n* = 164	*n* = 71
Median 7–9/consensus^a^	119	31
Median 4–6/consensus	24	14
Median 1–3/consensus	8	11
Median 7–9/disagreement	2	2
Median 4–6/disagreement	11	11
Median 1–3/disagreement	0	2

Note: The statements of iteration 1 were rated dichotomous or on a 1–5 Likert scale and could not be analysed using the RAND/UCLA criteria. Therefore, this iteration has been excluded from this table. ^a^Only statements with median 7–9/consensus were copied into the guideline.

### Content of the statements/practice guideline

The full version of the practice guideline is attached as supplementary file (supplement 1). The guideline addresses five main themes of which the main issues are described below (Table 3). Some more and detailed information about the themes were collected in three attachments about (1) threshold at which APs can be prescribed, (2) assessment prior to prescription, and (3) care and treatment plan. The statements of the attachments were also a result of the Delphi process.

**Table 3.** Themes and highlights of statements addressed in the practice guideline for antipsychotic prescription in dementia patients residing in long-term care

1. General prescription stipulations.
• Antipsychotics should never be used as a first-line approach. Non-pharmacological interventions should be tried first. The benefits should be expected to outweigh the adverse events.
• APs should only be prescribed in
(a) symptoms caused by underlying psychotic disorder that causes severe distress to patient/risk to others,
(b) in non-psychotic patients in an extreme and acute situation with risk i.e. severe and harmful physical aggression to oneself or other, severe physical exhaustion, and severe eating/drinking disorders with a risk of malnourishment or dehydration.
• The behavior is not caused by another somatic disorder (such as pain, infection, hunger, constipation) or psychiatric disorder (anxiety/depression),
• only antipsychotics with proven evidence should be prescribed,
• start low, go slow.
2. Assessment prior to prescription.
• Investigation of underlying syndromes, neurological, psychiatric, environmental (interaction) factors.
• Assessment of medical state and risk (cardiovascular and subtype of dementia (Lewy Body/Parkinson) and symptoms (motor symptoms, cardiac arrhythmias, orthostatic hypotension, urine retention).
• ECG should be carried out in patients with history of cardiovascular diseases, cardiac arrhythmia, and combination of medication that prolong QT-interval.
3. Care and treatment plan.
• Use APs always in combination with non-pharmacological and preventive measure aimed at increasing carers competence.
• Care and treatment plan should draw expertise form multidisciplinary team/with regular consultation.
• Family caregiver should be informed and consulted throughout treatment and discontinuation.
• Improvement and lack of improvement should be included as a clinical criterion for modifying care and treatment plan.
4. Discontinuation.
• Discontinuation should be a standard principle as part of a withdrawal plan.
• If APs are prescribed for sedative purposes, drug should be withdrawn when situation has calmed down.
• Discontinuation through tapering rather than immediate discontinuation unless Malign Neuroleptic Syndrome, cardiovascular complication, infections, severe side effect at low dose.
5. Long-term treatment (> 12 weeks).
• Long-term antipsychotic treatment is only acceptable in patients with
○ long history or high severity of psychotics/concurrent schizophrenia,
○ at least two unsuccessful discontinuation attempts + psychosocial interventions has been shown not to be effective + alternative medication is not available/has been shown ineffective/expected to cause severe adverse events.
• Restarting can be acceptable – under supervision of a specialist – in extreme situation in case of
○ recurrence of severe symptoms after withdrawal resulting in risk/distress that had previously improved with AP treatment,
○ recurrence of severe symptoms after withdrawal if withdrawal was before completing a 12-week course,
○ a distinct separate new episode.

#### General prescription stipulations

In the guideline, a distinction was made between prescribing an AP for treatment of severe symptoms (physical or severe verbal aggression or agitation or severely distressing anxiety) that are caused by an underlying psychotic disorder and prescription for sedative purposes in extreme and acute situations without psychosis. In the former case, APs should be given as long as this is needed for the treatment of psychosis, in the latter prescription should be withdrawn when the situation has calmed down. APs for an underlying psychotic disorder can only be prescribed in case of severe continuous distress affecting quality of life of the patient, family caregiver, or other patients; the behavior is not caused by another somatic disorder (such as pain, infection, hunger, constipation) or psychiatric disorder (anxiety/depression); moreover, psychosocial interventions have been tried without success and if the benefit is expected to outweigh the adverse events. AP drug prescription might be justified in case of an extreme situation (also without psychosis), if the behavior is causing acute and tangible risks to patient or other. Symptoms that may indicate an extreme situation are severe and harmful physical aggression, severe physical exhaustion, and severe eating/drinking disorders with a risk of malnourishment (weight loss) or dehydration.

#### Assessment prior to prescription

Before prescription of APs, proper assessment should be carried out of the target symptom(s) that warrant treatment, underlying medical causative factors (pain, infection, hunger, constipation), other psychiatric co-morbidities (depression, anxiety, sleep disorders, and delirium), social factors; and risk groups that may affect the decision to prescribe APs or not in the first place (cardiovascular diseases, cardiac arrhythmia, Lewy Body Dementia (LBD), and Parkinson's disease). Baseline assessment of motor symptoms, cardiac symptoms, orthostatic hypotension, and urinary retention that could be mixed up with side effects, should be carried out. Electrocardiogram was only deemed necessary in patients with a history of cardiovascular disease (including cardiac arrhythmias) or in patients with other medication that can prolong QT-interval.

#### Care and treatment plan/family involvement

AP treatment should always be combined with non-pharmacological interventions and preventive measures aimed at increasing caregivers competence to deal with NPS. The AP prescription should be part of the care and treatment plan which should include a further definition and specification of target symptom, associated risks and distress, treatment objective, how and when improvements and adverse events should be monitored. The patient (if relevant) and primary family caregiver should be consulted in the critical phases of treatment: before treatment, during monitoring, discontinuation and in case of long-term treatment. The most responsible clinician, rather than the nurse’s, should actively discuss the care and treatment plan with the family caregiver. This is usually the general practitioner, but depending on the way care is organized on a local or national level, this could be the elderly care physician or the consulting old-age psychiatrist. The treatment plan should also include routines for multidisciplinary consultation from the most responsible doctor, other consulting clinicians, nurse, relevant (in)formal caregiver. This team is responsible for monitoring AP treatment. An old-age psychiatrist should be consulted in severe cases that cannot be solved by the responsible physician.

#### Discontinuation

In case of prescription for psychosis and for sedative purposes, APs should be discontinued when the NPS (partly) resolve or the acute situation has calmed down. Discontinuation should be performed through tapering, to be able to monitor any relapse of symptoms, unless in cases of malign neuroleptic syndrome, cardiovascular events, infection or severe side effects (when the dose is low). In case of lack of improvement, the dose should be increased until side effects appear, and continued for a period of 4 weeks. If no improvement is observed after this period, APs should be withdrawn through tapering. Also, the diagnosis, target symptoms and treatment goals should be reviewed and alternative interventions or other agents should be considered.

#### Long-term treatment (>12 weeks)

Treatment of APs should not exceed 12 weeks. Longer treatment is only acceptable in cases of psychosis or schizophrenia or justified for the treatment of severe symptoms associated with dementia if two discontinuation attempts did not turn out to be successful, and psychosocial interventions or alternative psychotropic medication has been shown ineffective. Long-term treatment should always be handled by a specialist.

## Discussion

With the robust process of modified Delphi technique along with focus groups, that involved a large number of scientific, clinical, and consumer experts, we ended up with a set of 150 statements that were incorporated into a practice guideline, and that is ready to be used by professionals working with people with dementia in long-term care. The practice guideline provides clinicians key practical information that is lacking in current guidelines.

### Discussion of the guideline content

We were able to answer some key topics that are not sufficiently addressed in other practice guidelines.

#### A more refined definition of prescription indication and threshold to prescribe APs in agitation, aggression and psychosis

In this guideline, a differentiation was made for AP as treatment for psychosis and as sedation to cope with aggression but only in extreme or acute specified situations for a relatively shorter period. This differentiation is novel, since current clinical trials and practice guidelines do not distinguish between the two indications. The AAGS/AGP guideline (AAGS/AGP, 2003) only allows APs in behavior associated with psychosis; the Nice guideline (NICE guideline 42) allows medication in psychosis and/or agitation; the Canadian guideline (Canadian Guideline for senior mental health, 2006) sets the indication to behavior with or without psychosis but in the latter case not only restricted to acute circumstances. Only the expert panel of the DICE approach seems to somewhat distinguish two indications by stating that psychotropics may be prescribed for psychosis causing (potential to) harm and aggression causing risk to self or others (Kales *et al*., 2014). Although, there is some evidence for APs in the reduction of aggressive symptoms without psychosis (Ballard *et al.*, 2009b), experts evidently believe that the prescription of APs in aggression without underlying psychosis is more sedation rather than symptom resolution through treatment. That is also the reason why the threshold for prescription in non-psychotic acute aggression is advised to be higher than in aggression associated with psychosis. This differentiation among symptoms like aggression, agitation, and psychosis is in line with the more syndromal approach of diagnosing NPS (Lyketsos, 2007). The differentiation between psychosis and acute phase of aggression would also imply that the treatment duration for acute reasons (in non-psychotic extreme aggression) may be shorter than for the treatment of psychosis. When the situation has calmed down, there is no place for APs and there will be time to consider other (non-) pharmacological management of aggression/agitation.

#### A broader description of risk factors that should be considered before prescription

Before starting AP treatment, a thorough assessment of underlying syndromes, medical state, and risk factors should be carried out, which is in line with the AGS/AAGP paper (AGS/AAGP, 2003) and the US DICE approach (Kales *et al*., 2014). Increasingly important is the careful monitoring of pain as a possible (Pieper *et al.*, 2013), under-recognized (De Souto Barreto *et al.*, 2013) and treatable (Husebo *et al.*, 2014) underlying medical factor, which is also acknowledged in the Nice guideline (NICE guideline 42), and the US DICE approach (Kales *et al*., 2014). The novelty of the present guideline is the focus on prior assessment of symptoms that may be recognized as adverse effects (urine retention), and the clear focus on prior assessment of cardiovascular risk factors. In the focus groups, there was some discussion on the necessity of making an electrocardiogram to measure the QT-interval. Since the pre-test risk of prolonged QT interval in the general population is low, experts consider carrying out electrocardiogram in all patients before AP treatment not feasible and not efficient, but only in a subgroup of patients with higher pre-treatment risk with a history of cardiovascular diseases, cardiac arrhythmia, and a combination of medications that prolong QT-interval, which may include among others citalopram, escitalopram, methadone, ondansetron, and azithromycine (Trinkley *et al.*, 2013). Not only LBD, but Parkinson dementia (PDD) was also recognized as a risk factor for severe motor adverse events from APs. So recognition of LBD/PDD is vital before AP drug prescription.

#### Circumstances at which APs should be stopped or tapered

Practical advice on discontinuation is very necessary, given the reports of prolonged AP drug use in nursing homes (Wetzels *et al.*, 2011). Although there are numerous stop trials, there is no indication whether immediate discontinuation (Ballard *et al.*, 2009a) or tapering (Devanand *et al.*, 2012) should be preferable over the other. In this guideline, experts favor tapering of APs, also to monitor any symptom relapse at lower dosage.

#### Providing specific criteria for justifying long-term treatment

Although the general advice is to discontinue APs after 12 weeks in agitation or psychosis associated with dementia, there may be room for prolonged treatment in some specific circumstances. Although, in general, prolonged AP use may not be effective (Schneider, 2006), there is some conflicting evidence of its long-term efficacy. Although, stopping APs has proven to be feasible (Ruths *et al.*, 2008; Ballard *et al.*, 2009a; Declerq *et al.*, 2013), there may be subgroups of patients with high level of NPS (Ballard *et al.*, 2009a) or patients with previous response to risperidone (Devanand *et al.*, 2012) in which stopping after long-term treatment is associated with a relapse of symptoms. The present guideline is stricter in the maximum prescription duration (12 weeks instead of 6 months in the AGS/AAGPS guideline), but at the same time allow practitioners to prescribe APs for a longer period of time under strict predefined circumstances, although long-term prescription requires active involvement/supervision of a specialist (e.g. old age psychiatrist).

#### A broader involvement of multidisciplinary expertise and the role of the family caregiver in the process of AP prescription

Long-term care for people with dementia is multidisciplinary care. It is in this context that AP drug prescription should be initiated, monitored, and tapered. Since AP prescription should be considered in concert with psychosocial interventions, important decisions should be made in a multidisciplinary context and if necessary with external expertise.

In the focus group of consumer experts, the role of the family caregiver was considered very important not only in the decision to prescribe APs but also in case of dose change and stopping. Also the monitoring plan should be discussed with the family. Since prescription of APs is a medical decision, the physician rather than the nurse should discuss the care plan with the family. Current practice shows that only 62% of the caregivers consented the prescription of AP treatment and 84% of those find their opinion sufficiently weighted by the physician in the decision to prescribe AP (Cornegé-Blokland *et al.*, 2012). There is clearly room for improvement. The focus group did not discuss the nature of decision making (informed, consent, shared decision making).

### Implications for clinical practice/advices for implementation

This practice guideline reveals novel components that are expected to move practice beyond current guidelines. Scientific guidelines, although robustly weighing efficacy and risks of psychotropic drugs in clinical trials or meta-analyses, fail to address under which circumstances drugs should be prescribed, tapered, and under which circumstances long-term treatment is justified, since no studies on this specific conditions are conducted. Clinicians lack the step from “what to prescribe” towards “how to prescribe” which is not addressed by scientific guidelines. Thus, specifying indications for APs drug prescription and conditions for tapering/stopping and long-term treatment could help to lower unnecessary high prescription rates. Also, the recommendations about what risks factors and adverse effects should be assessed and monitored is expected contribute to a safer AP drug use. Another key issue is the consultation of family caregiver in all stages of prescription. The active involvement of the family caregiver as a legal representative – especially in a shared-decision making model – could contribute to a more careful consideration of AP drug prescription and prevent long-term drug use.

This guideline provides useful tools to bridge the gap between scientific guidelines and clinical practice. Distribution of the guideline through national Alzheimer's organization and bodies representing general physician’s, old age psychiatrists, geriatricians, and elderly care physician's would facilitate implementation of the guideline. The implementation of this guideline by clinicians is expected to improve the quality of psychotropic drug prescription.

### Strengths and limitations

We want to emphasize that the practice guideline, that has been produced by cooperation between experts in UK, Norway, and the Netherlands, can be used in Western European and perhaps other developed countries, but may not be generalized to other countries in the world. A strength of this guideline is the extensive underlying procedure in which not only scientific and clinical experts but also consumer experts were able to reach agreement. Furthermore, the novel approach of a combination of the modified Delphi process and focus group has added value for three reasons. First, rather than just forcing the experts to agree or disagree with pre-defined statements, they were actively involved in specifying the statements so that more consensus could be achieved. We considered this an important modification, for the same reasons that have been put forward by critics of traditional Delphi, namely that the method does not allow respondents to discuss issues raised or to elaborate on their views (Goodman, 1987; Walker and Selfe, 1996). An interaction between experts can enhance complex decision-making processes and clarify language and recommendations (Vakil, 2011). Instead of excluding the initial statement that cannot be agreed upon from the guideline, as would be the normal procedure in the Delphi method, we re-formulated the recommendation to ensure that it was both specific enough to be clinically relevant, and supported by the consensus view of a large pool of experts. Second, we adopted a method for calculating consensus that accounted for the dispersion of ratings among the respondents as well as the internal symmetry between ratings. Third, we combined different sources of experience – scientific, clinical, and consumer-based experience, and included experts from various professions. By including consumer experts, we also integrated the patient and caregiver perspective into the study, as opposed to restricting patient involvement to only reflecting on the final outcome. This gave the statements a strong support-base, which increases the legitimacy of the final recommendations. A limitation is the relative low number of consumer experts responding to the second questionnaire (*n* = 14) and participating in the focus group (*n* = 9), which may result in sampling error. Moreover, the focus group is only organized in the UK, due to practical reasons, which may limit generalizability of the caregiver perspective to other countries.

## Conclusion

With this practice guideline, we were able to bridge the gap between scientific evidence (as formulated in the scientific guidelines) and daily practice. Although the guideline is not a substitute for evidence-based guidelines, it provides an additional level of detail to inform clinical practice for further clinical decision making. The method has led to a formal consensus that uniquely combined patients as well as clinicians’ views. Most important, the modified Delphi process turned out to have added value because it contributed to resolution of several gaps that were unanswered or not addressed in previous practice guidelines. This method could also be used by producing other practice baseline guidelines for other patient groups.

We recommend that this guideline is translated into national languages in Western Europe, disseminated and implemented into clinical practice. This may help physicians working with people with dementia in long-term care to reduce inappropriate AP drug prescription.

## Conflict of interest

Clive Ballard has received grant/research support from Acadia and Lundbeck Pharmaceuticals, and speakers honoraria from Novartis, Acadia, Otsuka, Roche, Orion, Heptares, Bial, Napp, and Bristol-Myer Squibb Pharmaceuticals.

All other authors have no conflict of interest to declare.

## Description of the authors’ roles

Alice Johansson is the primary researcher, organized the Delphi procedure, data collection and analysis. Sytse Zuidema, Raymond Koopmans, and Clive Ballard developed the idea of the study. Matt Murray, Clive Ballard, and Alistair Burns have a role in setting up the focus group for consumer experts. Geir Selbaek organized the focus group in Norway. Alice Johansson, Sytse Zuidema, Raymond Koopmans, Clive Ballard, and Geir Selbaek contributed to the data analysis and to discuss the results. All authors had a contribution in writing the paper.

## Supplementary material

For supplementary material accompanying this paper visit http://dx.doi.org/10.1017/S1041610215000745. click here to view supplementary material
