# Supplementary material for: A consensus guideline for antipsychotic drug use for dementia in care homes. Bridging the gap between scientific evidence and clinical practice
Source: Int Psychogeriatr. 2015 Jun 10;27(11):1849–59. doi: 10.1017/S1041610215000745 (PMC4582430; doi:10.1017/S1041610215000745)
Supplement: Supplementary file 1 [file S1041610215000745sup001.docx]

SUPPLEMENT 1: (Supplementary Material for online publication)

# Antipsychotic drug prescription for patients with dementia in long-term care. A practice guideline for physicians and caregivers

## About this guideline

This guideline offers guidance for appropriate treatment with antipsychotics in clients in long-term care settings with neuropsychiatric symptoms due to dementia. The guideline consists of a number of key principles that should be adhered to in different phases of a treatment. The recommendations are the result of an international collaboration between UK, Norway and the Netherlands, funded by Dutch Alzheimer’s Association (WE.03-2010-01) and UK Alzheimer Society (grant nr.145). The guideline builds on aggregated up to date insights, experiences, and expert opinions from scientific and clinical experts throughout the world, with in-depth contribution from experts in the Netherlands, Norway and the UK, and from caregivers in the UK. The results were retrieved through a Delphi based consensus procedure in combination with focus groups, aimed at identifying areas of consensus and disagreement and to agree on a number of best practices.

The guideline is structured into five sub-sections: (1) General prescription stipulations; (2) Assessments prior to prescription; (3) Care and treatment plan; (4) Discontinuation; and (5) Long-term treatment. Three attachments follows with the guideline: Attachment 1 provides guidance on how to define the threshold at which treatment with antipsychotics may become appropriate. Attachment 2 gives a review of the specific assessments that should be carried out prior to prescription. Attachment 3 gives a review of what a care-and treatment plan should include, including a list of the most important medical risk factors to be monitored and routines in case of improvement/lack of improvement in an on-going care- and treatment plan*.*

Advisory Group: Alice Johansson, Clive Ballard, Geir Selbaek, Matt Murray, Alistair Burns, Raymond Koopmans, Sytse Zuidema.

### General prescription stipulations

Antipsychotics should never be used as a first-line approach. Non-pharmacological interventions should always be tried first and other pharmacological interventions should be considered.

Antipsychotics should only be prescribed to treat neuropsychiatric symptoms that are caused by an underlying psychotic disorder (e.g. paranoia; bi-polar disorder; delusions or hallucinations associated with dementia) ***AND*** that cause severe distress to the patient or risk of danger to other patients (see attach. 1).

Antipsychotics should not be prescribed for treatment of behaviours not caused by an underlying psychosis

Only in extreme and acute situations, where a person´s behaviour cause tangible and immediate risk of serious physical harm or death to others, antipsychotics may be used for sedative purposes, even when there is no underlying psychotic disorder. In such cases, there must be a risk of tangible physical harm to the patient self or others and the prescription should be withdrawn immediately, when the situation has calmed down.

The choice of appropriate antipsychotic agents should be based on a careful assessment of the expected efficacy, adverse events, and side effects.

Only antipsychotics with proven evidence of efficacy should be prescribed.

Antipsychotic treatment should start with a low dose as standard, to be increased if appropriate.

### Assessments prior to prescription

To avoid inappropriate treatment with antipsychotic drugs and to enable subsequent assessments of the effects of the treatment, prescription should always be preceded by:

- Proper investigation and documentation of underlying causative syndromes or factors (neurological, psychiatric, psychological, environmental, structural factors, interaction problems) (see attach 2)
- Assessment, documentation and analysis of the symptom
- Assessment and documentation of the patients’ medical condition and associated risks.

If prescribed in an acute situation, assessments should be carried out once the situation has calmed down.

Particular risk factors to be considered are:

- Cardiovascular diseases
- Cardiac arrhythmia
- Cases where patients use medication that can prolong the QT-interval
- Lewy Body Dementia
- Parkinson’ s disease.

To rule out that potential symptoms that may appear later, are associated with the antipsychotic treatment, the following assessments should be carried out at the very least:

- Motor symptoms
- Cardiac arrhythmias
- Orthostatic hypotension
- Urine retention.

ECG assessment should be carried out for patients with or with a history of:

- Cardiovascular diseases
- Cardiac arrhythmia
- Medication that can prolong the QT-interval.

### Care and treatment plan

Antipsychotic drugs should only be prescribed as part of a comprehensive care and treatment plan which should be carefully tailored to the individual patients’ needs..

Antipsychotic treatment should always be combined with non-pharmacological interventions and preventive measures aimed at increasing caregivers competence to deal with neuropsychiatric symptoms.

The care and treatment plan should draw on expertise from a multi-disciplinary team

There should be systems and routines in place for regular consultation meetings including the prescriber, nurse, and people who know the person well.

The family caregiver should be informed and consulted regularly throughout the treatment period.

The effects of the treatment with antipsychotics and medical risk factors, adverse events and side effects should be regularly and carefully monitored.

Improvement/ lack of improvement should be included as a clinical criteria for modifying the care and treatment plan.

### Discontinuation

Neuropsychiatric symptoms are often temporary and may disappear or change character as the disease deteriorates. Therefore, discontinuation should be the standard principle, even in case of psychotic disorders.

Discontinuation and changes in treatment plan should be performed according to a withdrawal plan. If prescribed for sedative purposes to prevent danger, the drug should be withdrawn once the situation has calmed down and be followed by proper assessments and alternative interventions.

Discontinuation should in principle be performed through tapering, rather than immediate discontinuation, unless in case of:

- Malign Neuroleptic Syndrome,
- Cardiovascular phenomena (arrhythmia, cardiac failure or hypotension),
- Infection related to the treatment,
- Severe side effects when the dose is low.

Discontinuation should be followed up through monitoring.

Discontinuation should be carried out in close joint working with all relevant professionals involved in the care of the patient. Care staff should be offered support in this process.

### Long-term treatment with antipsychotics

Treatment with antipsychotics should in principle not exceed 12 weeks. Treatment longer than 12 weeks is only acceptable if:

- The patient has a long history and/or high severity of psychosis *OR*
- Antipsychotics are necessary to treat a concurrent disorder e.g. schizophrenia

Antipsychotics for longer than 12 weeks is only justified *IF*:

- An attempt to discontinue treatment with antipsychotics has been unsuccessful on at least two occasions AND
- Psychosocial care/interventions has been shown not to be effective AND
- Alternative medication is not available, has been shown ineffective, or is expected to cause severe adverse events

Restarting antipsychotic treatment after discontinuation can be acceptable in extreme circumstances, if no major safety concerns had arisen during previous periods of treatment, in cases of:

- Recurrence of severe symptoms after withdrawal resulting in risk / distress that had previously improved with anti-psychotic treatment
- Recurrence of severe symptoms after withdrawal, if withdrawal was before completing a 12-week course
- A distinct and separate occurrence of a new episode of symptoms after a prolonged period without symptoms
- Long-term treatment with antipsychotics in people with dementia should always be handled by a specialist.

**Attachment 1: Defining the threshold at which antipsychotics can be prescribed**

Symptoms that may justify treatment with antipsychotics, *IF* they are related to a psychotic disorder, are:

- Severe physical aggression
- Severe *v*erbal aggression (e.g. shouting, cursing)
- Severe verbal agitation (e.g. vocal agitation, demanding behaviour)
- Severely distressing anxiety

**A**ntipsychotics should *not* be prescribed for treatment of:

- Hyper sexuality / inappropriate behaviour
- Apathy
- Verbal aggression (e.g. shouting, cursing) associated with dementia, without underlying psychosis
- Verbal agitation (e.g. vocal agitation, demanding behaviour) associated with dementia, without underlying psychosis

**Specific definition when antipsychotics may be prescribed in case of an underlying psychotic disorder**

1. The patient is suffering from severe *and* continuous distress, without any relief, and with a severe negative effect on the quality of life *AND/OR*
2. The behaviour is causing severe *and* continuous distress, without any relief, to other patients or family caregiver *AND*
3. The benefits of antipsychotic treatment is expected to outweigh the adverse effects/ events *AND*
4. The behaviour is not caused by an underlying medical or somatic disorder (e.g. pain, infection, hunger, defecation/constipation, sleeping disturbances) *AND*
5. The behaviour is not caused by anxiety disorder or depression *AND*
6. The behaviour is not caused by psychosocial factors or factors in the environment (e.g. interaction with staff or other patients) *AND*
7. Psychosocial and environmental interventions have been tried without any success

**Definition of the threshold for an acute extreme situation**

1. The behaviour is causing acute and tangible physical harm to patient self, *AND/OR*
2. The behaviour is causing acute and tangible risk of severe physical harm to others *AND*
3. Other non-antipsychotic sedative medicines are expected to cause more risks or are less likely to be effective than antipsychotics

Symptoms that may indicate an extreme acute situation, are:

- Severe and harmful physical aggression;
- Severe physical exhaustion;
- Severe eating/drinking disorders with a risk of malnourishment / dehydration.

**Note:** Some additional key principles that can guide the decision-making are:

- The threshold for prescription should be higher if the behaviour is not caused by an underlying psychotic disorder.
- The threshold for prescription should be higher if the problem identified is the distress to other patients or staff rather than distress to the patient him- or herself.
- The threshold for prescription should be higher if the behaviour is related to a specific social situation or environmental factor.

**Attachment 2: Assessments prior to prescription**

In all cases, a detailed assessment should be carried out, before prescribing antipsychotics (in case of prescription in an acute situation, assessment may be carried out when the situation has calmed down).

Assessments should be carried out through the means of, at least:

- Observation
- Physical examination
- Neurological examination
- Psychiatric examination
- Interview with patient (where possible), caregivers and other informants
- Available standardized assessment scales should be used (e.g. NPI, BEHAVE-AD, CMAI).

Assessment should include, at least:

Medical causative factors

- Cardiovascular diseases
- Infection (e.g. urinary tract or pneumonia)
- Pain
- Hunger
- Constipation

Alternative psychiatric co-morbidities

- Depression
- Anxiety
- Sleep disorders
- Delirium

Social or environmental factors

- Interaction with other residents, family and staff
- Physical factors such as space available in the care home, amount of stimuli, lack of privacy and autonomy

Manifestation of behaviour and associated risks

- Description of the behaviour, i.e. the manifestation, severity, and persistency of the target symptoms
- The consequences of the behaviour, i.e. the manifestation, severity, and frequency of risks and distress associated with the target symptom
- The cause of the behaviour, i.e. the cause of the target symptom including possible environmental triggers

***Risk groups***

Before prescribing antipsychotics, the following risk factors should be considered:

- Cardiovascular diseases
- Cardiac arrhythmia
- Cases where patients use medication that can prolong the QT-interval
- Lewy Body Dementia
- Parkinson’ s disease

Before initiating antipsychotic treatment, assessments of the following symptoms should be carried out, to rule out that symptoms that may appear later, are associated with antipsychotic treatment:

- Motor symptoms
- Cardiac arrhythmias
- Orthostatic hypotension
- Urine retention

ECG assessment should be carried out, before initiating any antipsychotic treatment, for the following risk groups

- Patients with a history of cardiovascular diseases
- Patients with a history of cardiac arrhythmia
- Patients with a combination of medication that can prolong the QT-interval

# Attachment 3: Care and treatment plan

The care and treatment plan should include, at least:

- Specification of target symptoms and the associated risks and distress
- Treatment objectives, i.e. definition of how the alleviation of the target symptom will reduce risks and distress and improve the patient’s quality of life
- Description of how improvements will be monitored
- Description of how medical risks, adverse events, and side effects will be monitored
- Psychosocial and environmental interventions
- Time to evaluate effects
- Routines for discontinuation
- Routines for consultation with at least the prescribing doctor, the nurse, relevant caregivers, and other informants who know the patient well.

*Consultation*

The care and treatment plan should draw on expertise from:

- A specialist
- A specialist nurse
- Other relevant expertise on indication

Consultation in an on-going care and treatment plan should be multidisciplinary and include all relevant parts involved in the care of the patient.

The key tasks of the consultation team should be to:

- Analyse behaviour and define target symptoms
- Develop care and treatment plan and define goals
- Support staff
- Monitor effects

An old age psychiatrist should be consulted, as second-line expert, in severe cases that cannot be solved by the responsible physician.

It should be standard in all care settings to provide on-going training for care personnel in how to deal with neuropsychiatric symptoms and about the risks and effects of antipsychotic drugs.

*Involvement of family*

The patient (if relevant) and the primary family caregiver should be actively consulted, when wishing to be so, in at least in the following phases of the treatment:

- Before prescribing antipsychotics.
- When monitoring improvement/lack of improvement in an on-going treatment plan
- If relevant, in case of treatment with antipsychotics longer than 12 weeks
- If relevant, before re-starting treatment with antipsychotics

It is the doctor’s responsibility, rather than the nurse’s to actively discuss the care and treatment plan with the family caregiver.

# *Monitoring*

Regular monitoring should include, at least:

Medical risk factors:

- Changes in cerebrovascular or cardiovascular risk status
- ECG (to enable identification of QT-interval changes or any other changes)
- Motor symptoms
- Cardiac arrhythmias
- Orthostatic hypotension
- Urine retention
- Somnolence/sedation

Effects:

- Effects on the severity and frequency of target symptoms and associated risks and distress for the client and others
- Effects on the decrease in the quality of life of the client
- Psycho-social and environmental factors that may have an impact on the target symptom
- Changes in response over time

Effects should also be monitored when dosage is stable.

It should be accounted for that improvements might be the result of spontaneous improvement of a simultaneous disorder.

Monitoring should be carried out by the means of, at least:

- Psychiatric examination by a physician
- Observation by a nurse
- Informal interview with patient (when possible) and caregivers

Available standardized monitoring scales should be used for monitoring.

Prescription should be automatically reviewed after a few weeks in the event of a change of care setting, but sooner in case of prescription for sedative purpose in an acute extreme situation.

Target symptoms should always be reviewed and defined in case of dose revision.

*Routines in case of improvement/ lack of improvement in an on-going care and treatment plan*

Improvement/ lack of improvement should be included as a clinical criteria for modifying the care and treatment plan.

In case of improvement in an on-going treatment plan, standard procedure should be to:

- Wait for stabilization before discontinuing treatment
- Withdraw antipsychotics after a period of **12 weeks without symptoms**

In case of lack of improvement in on-going treatment of psychosis with antipsychotics, the standard procedure should be to:

- Increase to maximum tolerated dose or until extrapyramidal or any other side effects appear
- Continue for a period of **+/- 4 weeks** with the increased/ maximum tolerated dose before drawing any conclusion of its efficacy (unless side effects appear*)*

If no improvement is seen after 4 weeks, standard procedure should be to:

- Withdraw antipsychotics through tapering
- Review the target symptoms / treatment goals
- Review the initial diagnosis

Review alternative intervention / other agents
